# Exogenous Melatonin Ameliorates the Negative Effect of Osmotic Stress in Human and Bovine Ovarian Stromal Cells

**DOI:** 10.3390/antiox11061054

**Published:** 2022-05-26

**Authors:** Ebrahim Asadi, Atefeh Najafi, James D Benson

**Affiliations:** Department of Biology, University of Saskatchewan, Saskatoon, SK S7N 5E2, Canada; eba352@usask.ca (E.A.); atn421@mail.usask.ca (A.N.)

**Keywords:** osmotic stress, ovarian stromal cells, melatonin, ROS, antioxidant, oxidative stress, female fertility preservation, cancer

## Abstract

Ovarian tissue cryopreservation transplantation (OTCT) is the most flexible option to preserve fertility in women and children with cancer. However, OTCT is associated with follicle loss and an accompanying short lifespan of the grafts. Cryopreservation-induced damage could be due to cryoprotective agent (CPA) toxicity and osmotic shock. Therefore, one way to avoid this damage is to maintain the cell volume within osmotic tolerance limits (OTLs). Here, we aimed to determine, for the first time, the OTLs of ovarian stromal cells (OSCs) and their relationship with reactive oxygen species (ROS) and mitochondrial respiratory chain activity (MRCA) of OSCs. We evaluated the effect of an optimal dose of melatonin on OTLs, viability, MRCA, ROS and total antioxidant capacity (TAC) of both human and bovine OSCs in plated and suspended cells. The OTLs of OSCs were between 200 and 375 mOsm/kg in bovine and between 150 and 500 mOsm/kg in human. Melatonin expands OTLs of OSCs. Furthermore, melatonin significantly reduced ROS and improved TAC, MRCA and viability. Due to the narrow osmotic window of OSCs, it is important to optimize the current protocols of OTCT to maintain enough alive stromal cells, which are necessary for follicle development and graft longevity. The addition of melatonin is a promising strategy for improved cryopreservation media.

## 1. Introduction

Advances in cancer diagnosis and treatment have reduced cancer mortality rates and expanded lifespans of cancer survivors [1,2]. However, cancer treatments, including chemotherapy and radiotherapy, can cause ovarian dysfunction and infertility because of follicular depletion [3,4]. With increasing cancer survivor life expectancy, survivor quality of life, including fertility preservation, has become progressively more important [5] as approximately 10% of female cancers occur at reproductive ages [6]. Recognized techniques for fertility preservation, such as oocyte or embryo cryopreservation, are not always appropriate for females with cancer since they need ovarian stimulation and time, causing delays in cancer treatments. Ovarian tissue cryopreservation and transplantation (OTCT) is not associated with these delays, and as such, is a promising method to preserve fertility for patients who cannot postpone cancer treatments. Moreover, it is the only option in prepubertal girls with cancer [7].

Although ovarian tissue cryopreservation has been used at many clinics around the world to preserve female fertility, and approximately 200 live births have been reported using this technique [8], there are still numerous challenges to be addressed. For example, large scale follicle loss during OTCT has been reported in many studies [9,10]. This may be due to a variety of injuries incurred during the cryopreservation process. For example, during cooling, ice formation is the main cause of cell damage, either directly (mechanically) or indirectly by alteration of the extracellular and, thus, intracellular chemical makeup [11]. Specifically, fast cooling is attributed to cell injury via intracellular ice formation, while slow cooling is associated with osmotic stress because of highly concentrated intra- and extracellular solutions [12,13]. Although it is believed that ice formation throughout the thawing procedure is less dangerous compared to ice forming during freezing procedures, studies have shown that it could cause harmful effects via two mechanisms; recrystallization, which is the annealing and growth of previously formed small ice crystals, and the icing of the solution after thawing [14,15]. Chemical cryoprotective agents (CPAs, e.g., DMSO or glycerol) protect cells and tissues against damage at sub-zero temperatures when at multimolal quantities. Thus, the first (and last) step of tissue cryopreservation is CPA equilibration, where cells and tissues undergo osmotically driven volume changes associated with damage mechanisms, including cell osmotic shock due to abrupt cell volume change, and intratissue stress due to the transport of water and solutes within the extracellular matrix [13,16]. To avoid osmotic and mechanically-induced damage, protocols that load and unload CPAs in a “stepwise” manner are an option to reduce or prevent these damages [17]. In fact, several successful equilibration approaches have been described [18,19,20,21]. For ovarian tissue, however, the majority of the cryopreservation protocols of ovarian tissue utilized do not use mathematically or biophysically informed CPA addition and unloading procedures [17]. In short, cell death during cryopreservation results from multiple factors, including cell volume excursions, ionic imbalances, chilling injury, cellular acidosis, membrane phase transitions and alterations, adenosine triphosphate (ATP) deprivation, protease (caspase) activation and free radical generation [22,23].

Worldwide, approximately 48.5 million couples (15% of couples) are estimated to be affected by infertility, and oxidative stress has been linked to infertility in both males and females [24,25]. Recently, a study showed that human sperm nuclear basic proteins (SNBP) played an important role in oxidative stress-induced DNA damage [26]. These alterations of SNBP, DNA and semen parameters might be possible to pass on to next generations [27]. Ovarian tissue cryopreservation is also associated with the generation of reactive oxygen species (ROS), the reduction of the antioxidant defense capacity and subsequent oxidative stress [28,29]. ROS can cause peroxidation of the polyunsaturated fatty acid (PUFA), DNA damage [30,31] and cellular apoptosis [32]. Specific to the structure of ovarian tissue, oxidative stress can damage follicles, including triggering primordial follicle activation and later burnout of the ovarian follicle reserve [33].

In many cell types, including sperm [34], oocytes [35], embryos [36] and stem cells [20], it has been shown that it is necessary to minimize damaging osmotic stress and the resultant cell volume changes via maintaining the cell volume within so-called osmotic tolerance limits (OTLs), the maximum volume excursions that cells can tolerate before irreversible damage happens, to optimize an effective cryopreservation protocol [37,38]. Moreover, previous studies in sperm have reported that osmotic stress can induce oxidative stress through the elevation of superoxide anion production and can, subsequently, cause cell damage [39,40]. The mechanism underlying this interaction is not well described; however, studies suggest that the activation of membrane-associated phospholipase A2 (PLA2) and NADPH oxidase could, subsequently, be a responsible pathway for superoxide anion production in response to osmotic stress [41,42]. This specific mechanism has not been explored beyond sperm, though the connection between cryopreservation and oxidative damage has been shown in oocytes, embryos, and ovarian tissue as a whole.

Cryopreservation-induced alterations in the volume of the cells in ovarian tissue has been suggested to be a source of cell damage and death, possibly based on the speed and intensity of volume changes [43]. The literature shows that there is wide variability among the tolerance of different cell types to osmotic stress, suggesting that the biology of the cell membrane, such as the membrane phospholipid structure, the activity of Na^+^/K^+^ ATPase, the membrane permeability to water, the lipid phase transition temperature, or ion and water channels, may determine the susceptibility of cells to osmotic stress, which alters their tolerance to CPA equilibration and freezing and thawing stress [37,44]. Most studies of cell tolerance to osmotic stress have been in homogenous populations of suspended cells. To our knowledge there have been few published reports of the osmotic tolerance of adherent cells [45,46], which may be more representative of the cell shape and intercellular connections that cells in tissues would experience. However, most tissues are heterogenous, and ovarian tissue is complex due to the fact that its cell types include follicles with sensitive granulosa–oocyte connections, and oocytes that are two to three orders of magnitude larger than their surroundings, stromal cells, granulosa cells, theca cells, etc.

During cryopreservation, all cells are exposed to the toxic effect of CPAs and the detrimental effects of freezing and warming steps [47], but a quantifiable link between ideal CPA equilibration and low toxicity in ovarian tissue cryopreservation is challenging to establish since each cell type likely has different optimal requirements for cryopreservation [47]. The main population of ovarian cortex cells is categorized as stromal cells (more than 80%) [48,49]. These cells play key roles in ovarian function and follicle development. For instance, different substances, including proteins and peptides secreted by ovarian stromal cells (OSCs), have been found to positively control the transition of follicles from the primordial to primary stages [50,51]. Moreover, extracellular matrix proteins, such as collagen, are produced by stromal cells for cellular and structural support and repair [52,53]. Another study showed that stromal cells were able to increase proliferation of the granulosa cells, estradiol and progesterone hormones production and prevent apoptosis in the in vitro co-culture system [54]. On the other hand, it is believed that factors produced by the granulosa cells of the active primary follicle are responsible for recruitment and/or differentiation of the surrounding stromal cells to thecal cells and these theca cells are responsible for providing androgens [51,55].

While the primordial follicles seem to be resistant to the detrimental effects of cryopreservation, and oocytes appear to be preserved after ovarian tissue cryopreservation, various investigations have found that the freezing–thawing method has a deleterious impact on stromal cells, especially when slow freezing methods are used [56,57]. Slow freezing is a class of methods where extracellular ice is encouraged and cooling rates are slow enough to facilitate dehydration and protection of the intracellular spaces and is the standard protocol for ovarian tissue cryopreservation in clinics. The negative effects of these protocols in stromal cells and ovarian tissues include empty areas in the extracellular matrix with disordered collagen and fibrin proteins and increased numbers of necrotic stromal cells with pyknotic and irregular-shaped nuclei, condensed chromatin, cytoplasmic vacuoles and plasma membrane lysis [56,58]. Moreover, the density of the stroma was reduced by both exposure of ovarian tissues to CPAs and cryopreservation procedures in goats and sheep [59]. Therefore, it seems that stromal cell density might play a significant role in follicular survival and growth either in the in vitro culture system or after transplantation. Previous studies have stated that the addition of exogenous antioxidants such as trolox, resveratrol, vitamin C and melatonin to the cryopreservation medium ameliorates some of the detrimental effects of cryopreservation in the reproductive cells and tissues [60,61,62,63,64]. A study showed that the addition of resveratrol to the freezing extenders protects boar sperm against ROS damage and improves the antioxidative defense system [64]. Another study reported that Trolox (water-soluble vitamin E) reduced apoptosis and improved DNA integrity of cryopreserved semen samples in normal and oligoozoospermic men [62]. Moreover, the supplementation of cryopreservation media with the combination of vitamin E and vitamin C had more protective effect against ROS and cold shock compared to vitamin E and vitamin C alone in cryopreserved sperm [63].

Another candidate antioxidant for protecting ovarian tissue during cryopreservation is melatonin (*N*-acetyl-5-methoxytryptamine), which is a highly lipophilic molecule, can easily cross the cell membranes and is mainly produced and secreted by the pineal gland [65], though it may also be produced in the ovary [66]. Melatonin plays a key role in regulating many physiological functions such as the circadian rhythm, reproductive cycle [67] and the immune system [68]. Follicular fluid contains melatonin, which is synthesized by the oocytes or taken from the blood stream [69,70]. Melatonin was reported as an efficient predictor of successful in vitro fertilization (IVF) outcomes for cryopreserved sperm, and melatonin in synergy with myo-inositol was able to improve oocyte and embryo quality in patients with polycystic ovarian syndrome [71]. Additionally, it can effectively reduce oxidative stress through a different mechanism by working as a scavenger of free radicals, upregulating the expression of antioxidant proteins and inhibiting lipid peroxidation and DNA damage [29]. In fact, melatonin is several times more powerful than vitamin E and C in defending cells and tissues against the detrimental effect of oxidative stress at an equivalent amount (mmol/kg) [72], and a previous study showed that melatonin and salicylic acid could reduce the negative effects of heat stress and increased antioxidant protein levels [73].

Despite the importance of stromal cells in ovarian function and follicle development, information about their behavior during the cryopreservation procedure is limited. The present study aimed to determine, for the first time, the OTLs of OSCs. We demonstrate that these limits are correlated with ROS production levels and mitochondrial respiratory chain activity (MRCA) of OSCs. This novel connection between osmotic stress and ROS lends itself to antioxidant mitigation. Thus, here, we evaluate the effect of melatonin as an antioxidant on osmotic sensitivity, viability, MRCA, ROS production levels and total antioxidant levels of both human and bovine OSCs, plated and suspended, to clarify the influence of cell–cell surface interaction on osmotic tolerance, we divided them in suspended and adherent groups to have better understanding about their reaction in tissue following exposer to osmotic stress. Our results provide fundamental information to improve current approaches for the cryopreservation of ovarian tissues.

## 2. Materials and Methods

### 2.1. Chemicals

All chemicals were purchased from the Thermo Fisher Company (Mississauga, ON, Canada) unless otherwise stated.

### 2.2. Ovarian Tissue Collection

#### 2.2.1. Bovine

Animal ethics approval was obtained from the University of Saskatchewan University Animal Care Committee (UACC; #024Exempt2021). Pairs of whole ovaries were obtained from bovine cows, aged from 14 to 18 months, at a local abattoir (*n* = 24). The samples were then placed in a sample container containing MEM medium supplemented with 25 mM HEPES, 1% streptomycin-penicillin G. Ovaries were subsequently transported to the laboratory on ice at 4 °C within 30 min. Under a laminar flow hood, the exterior of the ovaries was washed once with 75% alcohol and then three times in the fresh medium with supplements as described above.

#### 2.2.2. Human

Human ethics approval was obtained from the University of Saskatchewan Research Ethics Board (Bio-REB-3069). Human ovarian biopsies were collected from three patients (40 to 48 years old) who underwent an oophorectomy. Informed consent was obtained from patients. Ovarian tissues were retrieved by laparoscopy and transported to the laboratory on ice within 20 min in the above-mentioned fresh media.

### 2.3. Ovarian Tissue Processing and Stromal Cell Isolation Procedure

Under the laminar flow hood, following the removal of surface epithelium and medulla with surgical scissors and scalpel, the ovarian cortex was cut into small fragments of 1 × 1.5 × 0.5 mm. OSCs were isolated following the methods described by Soares et al. [74] with some modification. Briefly, ovarian cortical fragments were minced and incubated in 2 mL DMEM-F12 medium supplemented with DNase enzyme (0.02 mg/mL) and Liberase DH enzyme (0.08 mg/mL) (Roche Diagnostics, Mannheim, Germany) and 1% streptomycin-penicillin G at 37 °C and 5% CO_2_ for 45 min. The tissue was mechanically dispersed by pipetting digested tissue every 15 min. Following the incubation, an equal volume of cold DMEM-F12 medium supplemented with 10% fetal bovine serum (FBS) was added to inactivate the activity of the enzymes. Then, the digested cell suspension was consecutively filtered through sterilized 80, 40 and 11 µm nylon net filters (Millipore, Etobicoke, ON, Canada) and subsequently centrifuged at 500 g for 5 min. The pellet was then re-suspended in 5 mL of DMEM-F12 supplemented with L-glutamine, sodium bicarbonate 1% streptomycin-penicillin G and 10% FBS. Cells were then counted, and 1 × 10^6^ cells were loaded into a T75 culture flask. The cell culture flask was placed in an incubator at 37 °C and 5% CO_2_. The culture medium was exchanged with fresh culture medium every two days. When the confluency of cultured stromal cells reached approximately 80%, they were trypsinized with Trypsin-EDTA (0.25%) (Gibco, Waltham, MA, USA) to disperse the cells into suspension or for use in further experiments. After an initial viability assessment using trypan blue staining, the volume of the cell suspensions was adjusted to equalize the cell density in each well. The cell suspension was divided into two parts and each part was used for the different experiments. One part of the cell suspension was seeded into a 96-well plate at a density of 1 × 10^4^ cells per well and cultured for 2 days, which is when the cells reached an approximate confluency of 80% before experimentation. The second part was used for suspended cell OTL assessments in the concentration of 4 × 10^4^ cells per group.

### 2.4. Ovarian Stromal Cell Viability and Concentration after Trypsinization

The cell count and viability assessments were performed using trypan blue staining. Briefly, 100 µL of the stromal cell suspension was transferred to a microtube and incubated with an equal volume of 0.4% trypan blue solution for approximately 1 min at room temperature (RT). 10 µL of cell suspension was then loaded in a hemocytometer (counting chamber) and observed under a bright-field microscope.

### 2.5. Anisotonic Solutions Preparation

To evaluate OTLs, hypertonic and hypotonic solutions were prepared using distilled water, sucrose solution and isotonic culture medium. Hypotonic solutions were prepared by the addition of sterile distilled water to the isotonic culture media to produce solution osmolalities of 25, 50, 75, 100, 150, 200, 250 and 275 mOsm/kg. Hypertonic solutions were made by mixing sucrose solution with culture media to create solution osmolalities of 325, 350, 375, 400, 425, 450, 475, 500, 700 and 1000 mOsm/kg. The osmolalities of test solutions were measured using a freezing point depression osmometer (Fiske 2400 Multi-Sample Osmometer) and then sterilized by filtering through a 0.22 µm pore filter (Millipore, Etobicoke, ON, Canada).

### 2.6. Phase 1: Detection of OTLs of Bovine OSCs, Together with the ROS Production Levels and MRCA Activity after Exposure to the Different Range of Anisotonic Solutions

#### 2.6.1. Osmotic Sensitivity Assessment

The osmotic sensitivity of stromal cells was evaluated by the percentage of viable cells exposed to a range of anisotonic solutions compared to control groups (300 mOsm/kg) [75]. The viability was assessed using the calcein-AM cell viability kit (Thermo Fisher Scientific, Mississauga, ON, Canada; C3100MP). Each sample was tested in duplicate. For suspended cells, the cells suspension was centrifuged at 500 g for 5 min after each step to remove the supernatant and the pellet re-suspended in working solutions (as will be explained). Both adherent and suspended cells were exposed to different hypotonic and hypertonic solutions and incubated for 15 min at 37 °C and 5% CO_2_, followed by 5 min equilibration with isotonic culture media in the incubator. To measure the cell viability, the cells were washed with an isotonic culture medium and exposed with 2 µL calcein-AM and then returned to the incubator for 45 min at 37 °C and 5% CO_2_. As a negative control for calcein-AM (dead cells), cells were exposed to 3% formaldehyde for 30 min at 4 °C to induce cell death and subsequently subjected to the same wash procedures using the isotonic culture media. As a positive control for calcein-AM (live cells), cells were subjected to the same washing procedures using the isotonic culture media. To evaluate the background fluorescence of dye, calcein-AM was added to a subset of cell-free wells. All the fluorescence procedures were performed in a dark room or with covered plates or tubes. Calcein fluorescence intensity was then quantified using a plate reader (Varioskan Flash). The excitation and emission wavelengths were 485 nm and 530 nm, respectively. The obtained fluorescence intensity was normalized with the control group to calculate the percentage of viability in all experimental groups.

#### 2.6.2. Reactive Oxygen Species Levels Measurement

To detect intracellular ROS levels, 2′, 7′-dichlorofluorescein diacetate (DCFH-DA; Sigma-Aldrich, Oakville, ON, Canada; 287810) as a fluorescent oxidative dye was used [76]. Both adherent and suspended cells were incubated with DCFH-DA (25 μM) for 40 min at 37 °C and 5% CO_2_. After incubation, the formation of fluorescent dichlorofluorescein, as a product of DCFH-DA oxidation in the presence of ROS (primarily hydroperoxide), was measured by using a fluorescence microplate reader (Varioskan Flash) at excitation/emission wavelengths of 500/530 nm. Each sample was tested in duplicate, and results were expressed as the fluorescence intensity. As a negative control, buffer solution without DCFH-DA was added to the cells. As a positive control, cells were exposed to 500 μM H_2_O_2_ for 30 min. To assess the background fluorescence of dye, DCFH-DA was added to a subset of cell-free wells. All the fluorescence procedures were performed in a dark room or with covered plates or tubes.

#### 2.6.3. Mitochondrial Respiratory Chain Activity Measurement

To evaluate MRCA, resazurin (Sigma-Aldrich, Oakville, ON, Canada; R7017) as an indicator of cellular metabolic ability was used. Cells in all groups were subjected to resazurin (6 mM) for 60 min at 37 °C and 5% CO_2_. After the intracellular reduction in resazurin to resorufin, the fluorescence intensity was measured using a plate reader at 530 nm excitation and 590 nm emission. Two wells of each 96-well plate were allocated for each group. As a negative control, cells were exposed to 3% methanol for 30 min at 4 °C to induce cell death. As a positive control, cells were subjected to the isotonic culture media without the dye. To evaluate the background fluorescence of dye, resazurin was added to the cell-free wells. The resorufin-induced fluorescence intensity was normalized by the control group both in adherent and suspended cells. Then the reduction in MRCA was computed by the difference between the normalized results of the calcein-AM test and resazurin test in each group. Both assays are used to identify cell viability, but with different approaches. Therefore, the difference between normalized results shows the exact amount of reduction in MRCA. Since we seeded the same number of cells in both treatments, we expected to have the same number of viable cells after exposure to a similar osmotic condition [77].

### 2.7. Phase 2

#### Identification of Optimum Concentration of Melatonin

After measurement of the osmotic sensitivity of stromal cells, both suspended and adherent bovine OSCs were exposed to selected hypoosmotic (25 mOsm/kg and 150 mOsm/kg) and hyperosmotic (500 mOsm/kg and 1000 mOsm/kg) solutions containing concentrations of melatonin (MLT; 0 mM (control), 0.001 mM, 0.01 mM, 0.1 mM, 1 mM and 10 mM) (Sigma Aldrich, Burlington, MA, USA) and incubated for 15 min at 37 °C and 5% CO_2_, followed by a 5 min equilibration with isotonic culture media in the incubator. These concentrations of melatonin were selected based on previous studies [28,78]. To determine the optimal dose of melatonin, the cell viability and ROS production levels were evaluated in all groups.

### 2.8. Phase 3

After determination of the optimal dose of melatonin, both human and bovine OSCs were exposed to an isosmotic solution (25, 150, 500 and 1000 mOsm/kg) with or without supplementation with the optimal melatonin dose (0.1 mM). Then, the total antioxidant levels, ROS level, apoptosis, viability, cells proliferation rate and the MRCA were assessed in both species.

#### Total Antioxidant Capacity Measurement

The total antioxidant capacity (TAC) of the stromal cells in all groups was evaluated using the colorimetric Total Antioxidant Capacity Assay Kit (Abcam, Cambridge, UK; ab65329) according to the manufacturer’s protocol. Briefly, suspended and adherent cells in different groups were collected and washed in cold PBS. Then, cells lysed using ddH2O supplemented with 0.05% Triton and homogenized quickly. TAC was examined by incubating lysates with the Cu^2+^ working solution for 90 min on a shaker at room temperature. The small molecule and protein antioxidants convert Cu^2+^ ions to Cu^+^. Absorbance was measured using a microplate reader (Varioskan Flash) at 570 nm. TAC was calculated using a standard curve, and results were expressed as antioxidant concentration (mmol). Each sample was tested in duplicate. Trolox (6-hydroxy-2,5,7,8-tetramethylchroman-2-carboxylic acid) (0, 12, 24, 36, 48, 60 μL) was used as a standard to determine the Trolox equivalent capacity of the tested samples.

### 2.9. Statistical Analysis

SPSS software (version 23) was used to analyze the data. The data were tested for normality by using the Kolmogorov–Smirnov test. One-way analysis of variance was used to compare the mean values across the groups, followed by Turkey’s or Tamhane’s tests. Correlation analysis was conducted using Pearson’s r tests. The results are presented as the mean ± standard error. In the current study, statistical significance was set at *p* < 0.05.

## 3. Results

### 3.1. Osmotic Tolerance Limits (Phase 1)

**Figure 1:**
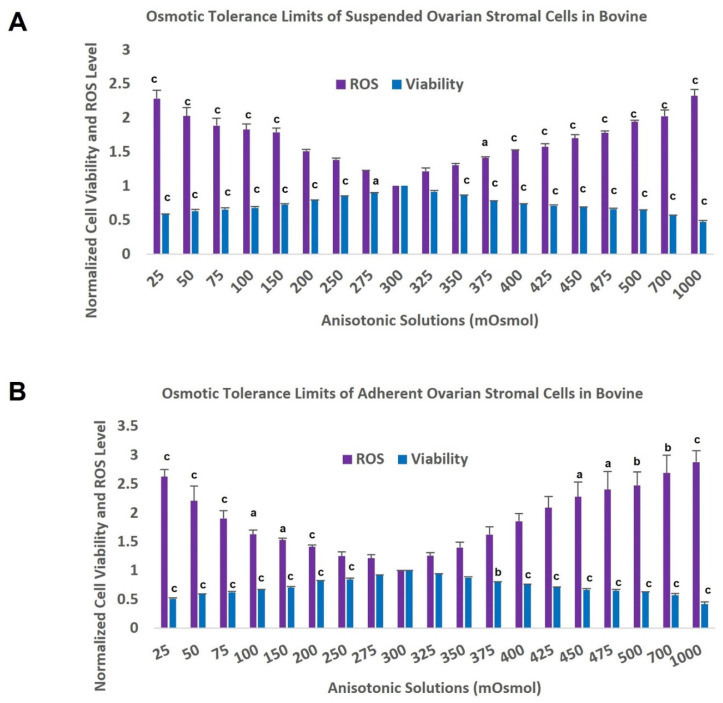
Osmotic tolerance limits of suspended (**A**) and adherent (**B**) bovine ovarian stromal cells using the comparison of percentage of viability and ROS levels after exposure to anisosmotic conditions. All data are normalized mean ± standard error of the mean (SEM). (a) *p* < 0.05 vs. 300 mOsm/kg group, (b) *p* ≤ 0.01 vs. 300 mOsm/kg group, (c) *p* ≤ 0.001 vs. 300 mOsm/kg group. (*n* = 8).

The effects of the anisotonic treatments on the viability of bovine OSCs, normalized to the isosmotic group (control group), and their relevant p value is shown in Figure 1. The upper and lower OTLs obtained was defined to be the osmolality at which approximately 80 percent of the cell population survived. In bovine tissue, our results showed that the OTLs of stromal cells was approximately between 200 and 375 mOsm/kg, both in suspended and adherent stromal cells, considering that the percentage of viable cells was 79% ± 0.3 and 78% ± 0.8 in suspended cells exposed to the hypo and hyper osmotic solutions, respectively, and 82% ± 1.4 and 79% ± 1.2 in adherent cells exposed to hypo and hyper osmotic solutions. In human tissue (phase 3), because of the small size of ovarian pieces, viability was evaluated in selected anisotonic solutions (25, 150, 500 and 1000 mOsm/kg), and the result was compared with the isotonic solution as well as the non-treated counterpart groups. Based on our results, the lower and upper OTLs of human stromal cells were approximately 150 and 500 mOsm/kg consecutively, with a cell viability of 80% ± 2 and 81% ± 1 in suspended cells exposed to the hypo and hyper osmotic solutions, respectively, and a viability of 79% ± 1 and 78% ± 3 in adherent cells exposed to hypo and hyper osmotic solutions (Table 1). In both species, a higher percentage of the stromal cells died as the solution concentration in which the cells were incubated deviated further from the isosmotic condition. A negative correlation was found between the deviation of osmolality from the isotonic solution into hypoosmotic solution and cell viability with a value of (r^2^ = −0.925, r = 0.962) for suspended cells and (r^2^ = −0.937, r = −0.968) for adherent stromal cells. This value showed significant correlation in hyperosmotic conditions, which was (r^2^ = −0.744, r = −0.863) and (r^2^ = −0.747, r = −0.864) for suspended and adherent stromal cells, respectively.

In humans, a significant correlation was observed between the degree of hypoosmolality and stromal cells viability, (r^2^ = −0.944, r = −0.972) for suspended stromal cells and (r^2^ = −0.971, r = −0.985) for adherent stromal cells. This correlation was also observed in hyperosmotic conditions, which was (r^2^ = −0.894, r = −0.946) and (r^2^ = −0.915, r = −0.956) for suspended and adherent stromal cells, sequentially (*p* < 0.05).

### 3.2. Oxidative Stress Measurement

ROS level results and their related p value in the bovine adherent and suspended stromal cells exposed to the range of anisotonic conditions are indicated in Figure 1. Regression analysis indicated that ROS levels were significantly increased when osmolality deviated from the isotonic condition compared with the control group. However, there was no significant difference in this value when the osmolality was between 200 and 350 mOsm/kg compared to the control group in suspended stromal cells and between 250 and 425 mOsm/kg in adherent stromal cells. In human OSCs, ROS levels were significantly enhanced in the selected anisotonic solutions both in suspended and adherent groups (Table 1). We observed significant positive correlation between osmolality changes from the isotonic condition into hypotonic conditions and ROS production levels in suspended stromal cells (r^2^ = 0.809, r = 0.900) and adherent stromal cells (r^2^ = 0.775, r = 0.880). In hyperosmotic conditions, positive correlation was observed between the degree of hyperosmolality in hyperosmotic conditions and the ROS level production, which was (r^2^ = 745, r = 0.863) and (r^2^ = 0.475, r = 0.689) for suspended and adherent stromal cells, respectively. In the human cells, the relation between hypo-osmolality conditions and ROS production were significant with a value of (r^2^ = 851, r = 0.923) for suspended cells. These variables showed positive correlation with a rate of (r^2^ = 963, r = 0.981) for adherent stromal cells. Our result showed that hyperosmolality is related significantly with ROS levels in suspended stromal cells (r^2^ = 937, r = 0.968) and adherent cells (r^2^ = 976, r = 0.988) (*p* < 0.05).

### 3.3. Mitochondrial Respiratory Chain Activity

**Figure 2:**
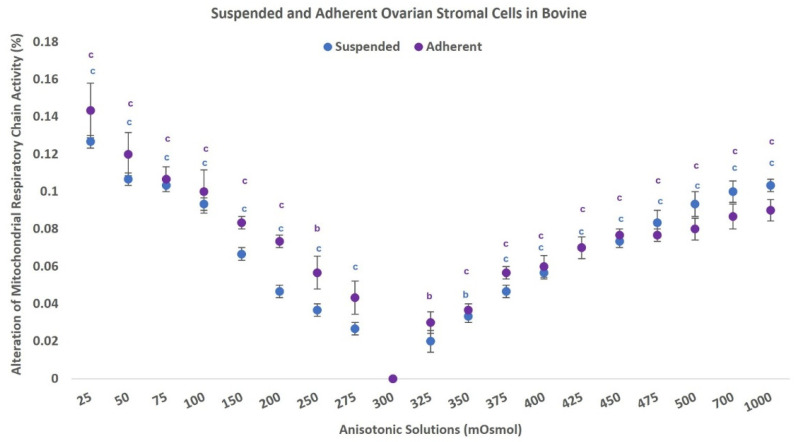
A comparison of percentage of alteration of respiratory chain activity in bovine ovarian stromal cells (suspended and adherent) after exposure to anisosmotic conditions. Each point represents the normalized mean ± standard error of the mean (SEM). Letters represent (b) *p* ≤ 0.01 vs. 300 mOsm/kg group, (c) *p* ≤ 0.001 vs. 300 mOsm/kg group (*n* = 8).

**Figure 3:**
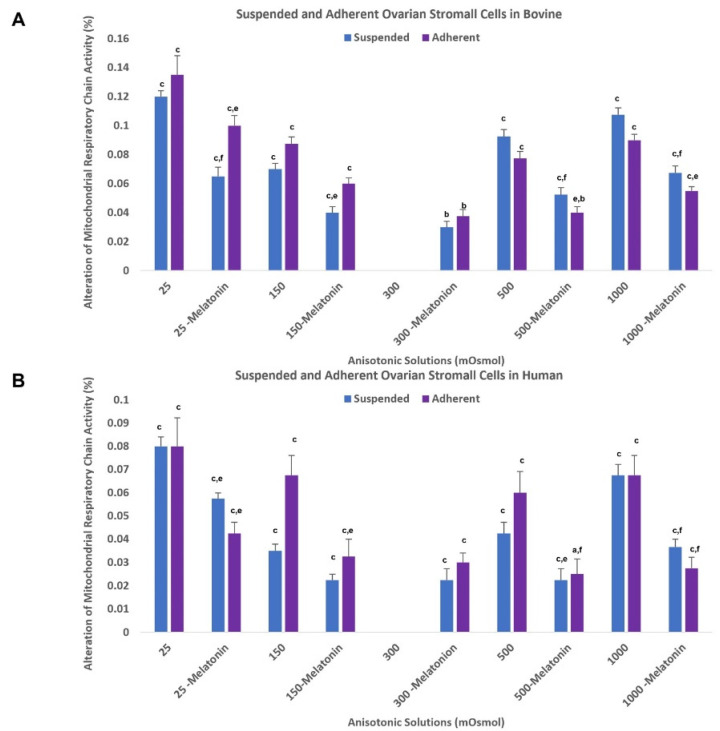
A comparison of percentage of alteration of respiratory chain activity in suspended and adherent ovarian stromal cells (bovine (*n* = 8) (**A**) and human (*n* = 3) (**B**)) after exposure to anisosmotic solutions supplemented with or without 0.1 mM melatonin. Each point represents the normalized mean ± standard error of the mean (SEM). (a) *p* < 0.05 vs. 300 mOsm/kg group, (b) *p* ≤ 0.01 vs. 300 mOsm/kg group, (c) *p* ≤ 0.001 vs. 300 mOsm/kg group, (e) *p* ≤ 0.01 vs. non-treated counterpart group, (f) *p* ≤ 0.001 vs. non-treated counterpart group.

The impacts of the hypo-osmolality or hyperosmolality conditions on MRCA of both adherent and suspended bovine OSCs with their p values are presented in Figure 2. The percentage of MRCA significantly decreased in both suspended and adherent stromal cells with deviation from isosmolality. For bovine cells in hypoosmotic conditions, MRC activity decreased to 14% and 12.5% in 25 mOsm/kg solutions in suspended and adherent cells, respectively. In hypertonic conditions, MRC activity decreased to 10% and 9% in a 1000 mOsm/kg solution for suspended and adherent stromal cells, respectively. These results demonstrate a negative significant correlation between the degree of hypo-osmolality and MRC activity in suspended (r^2^ = −0.970, r = −0.985) and adherent stromal cells (r^2^ = −0.849, r = −0.922), and these results demonstrate a significant correlation between the degree of hyperosmolality and MRC activity, which was (r^2^ = −0.568, r = −0.754) and (r^2^ = 459, r = −0.678) for suspended and adherent stromal cells, respectively. In human cells, our results showed a significant reduction in MRC activity in the selected anisotonic solutions compared to the control group (Figure 3B). Additionally, a significant negative correlation between hypo-osmolality degree and the MRCA was observed with the value of (r^2^ = −0.962, r = −0.981) for suspended cells and (r^2^ = −0.784, r = −0.886) for adherent cells. The deviation from isosmolality to the hyperosmotic condition showed significant negative correlation with the MRCA with the value of (r^2^ = 0.807, r = 0.898) for suspended cells and (r^2^ = −0.547, r = −0.740) for adherent cells (*p* < 0.05).

### 3.4. Optimal Concentration of Melatonin (Phase 2)

The impact of the different concentrations of melatonin on OSCs viability is presented in Table 2. Our results suggested that supplementation of anisotonic solutions with 0.1 mM of the melatonin (an optimal dose) can significantly improve the viability of both adherent and suspended cells in all isotonic solutions compared with their non-treated counterpart groups. Additionally, the treated groups with 10 mM melatonin showed a significant reduction in cell viability compared to the nontreated groups. To determine an effective dose of melatonin, we also evaluated intracellular ROS levels in all groups using DCFH-DA assay. Our results indicated that the treated group with 0.01- and 0.1-mM melatonin produced a significantly lower level of the ROS compared with one in the control groups; however, considering the vitality result, 0.1 mM melatonin was chosen as the optimal dose. Again, the 10 mM melatonin group showed a significantly higher level of ROS production in most of the treated groups compared with their control groups (*p* values are presented in Table 2).

### 3.5. Phase 3

#### 3.5.1. Effect of Optimal Dose of Melatonin on Ovarian Stromal Cells Osmotic Tolerance Limits (Cell Viability)

After incubation of ovarian adherent and suspended stromal cells with the above-mentioned chosen anisotonic solutions supplemented with the optimal dose of melatonin (0.1 mM), cell viability was evaluated both in human and bovine cells (Table 1). Based on our data, this optimal dose of melatonin for bovine cells was able to decrease the osmotic sensitivity of both adherent and suspended stromal cells. The lower OTL significantly decreased from 200 mOsm/kg to less than 150 mOsm/kg with 84% and 89% cell viability in suspended and adherent cells, respectively. The upper OTL was increased from 375 mOsm/kg to above 500 mOsm/kg with 85% and 90% viable cells in suspended and adherent cells, respectively. In human cells, the same trends were observed, where the lower OTL dramatically decreased from 150 mOsm/kg to approximately 25 mOsm/kg, with a measured viability of 76% and 75% in suspended and adherent groups, respectively. The upper OTL was increased from 500 mOsm/kg to nearly 1000 mOsm/kg with viability values of 75% for suspended cells and 73% for adherent cells (*p* values are presented in Table 1).

#### 3.5.2. Effect of Optimal Dose of Melatonin on Osmotic Stress-Induced ROS

As mentioned above, when stromal cells were subjected to hypotonic or hypertonic conditions, a significant increase in ROS generation was detected. However, the addition of 0.1 mOsm/kg melatonin to selected anisotonic solutions significantly reduced the osmotic stress-induced ROS levels in treated groups compared to control groups in both human and bovine OSCs. These results and their related *p* values are presented in Table 1.

#### 3.5.3. Effect of Optimal Dose of Melatonin on Mitochondrial Respiratory Chain Activity

Based on our data shown in Figure 3A, in bovine cells, an optimal dose of melatonin can significantly reduce MRC activity in the treated groups compared to the control groups. In human cells, the addition of 0.1 mM melatonin to anisotonic solutions can maintain MRCA well; however, this difference was not significant in some groups (*p* values are presented in Figure 3B).

#### 3.5.4. Effect of Optimal Dose of Melatonin on Total Antioxidant Capacity

**Figure 4:**
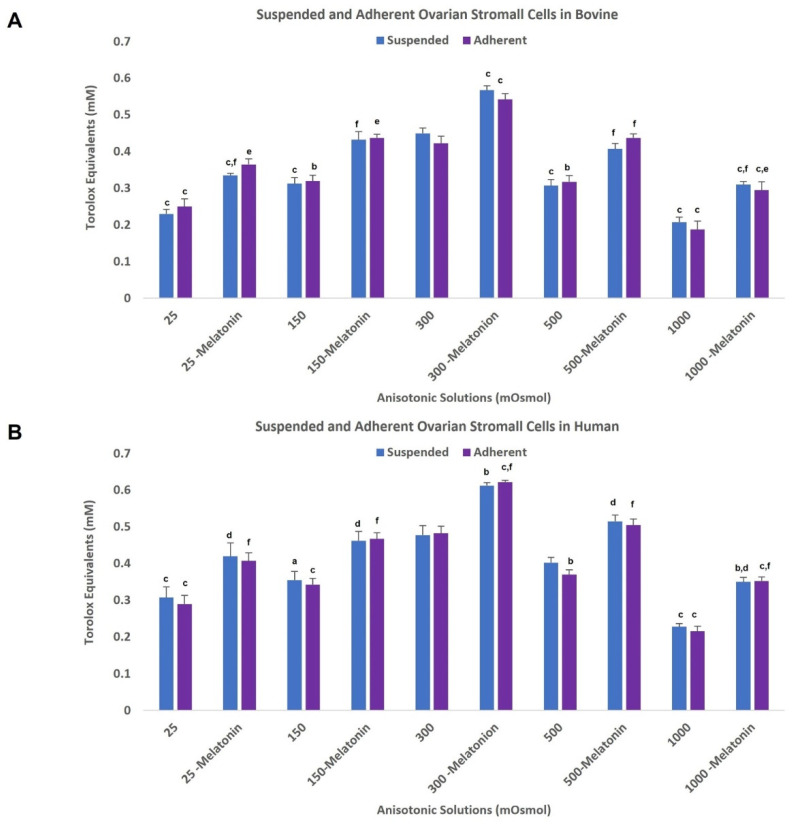
Total antioxidant capacity of suspended and adherent ovarian stromal cells in bovine (*n* = 8) (**A**) and human (*n* = 3) (**B**) after exposure to anisosmotic solutions supplemented with or without 0.1 mM of melatonin. All data are normalized mean ± standard error of the mean (SEM). (a) *p* < 0.05 vs. 300 mOsm/kg group, (b) *p* ≤ 0.01 vs. 300 mOsm/kg group, (c) *p* ≤ 0.001 vs. 300 mOsm/kg group, (d) *p* < 0.05 vs. non-treated counterpart group, (e) *p* ≤ 0.01 vs. non-treated counterpart group, (f) *p* ≤ 0.001 vs. non-treated counterpart group.

The result of TAC in all previously mentioned groups and their related *p* value are presented in Figure 4. A significant increase in the human and bovine OSCs antioxidant capacity was observed in the melatonin treated groups (0.1 mM) compared to control groups.

## 4. Discussion

In recent years, significant progress has been made toward successful OTCT. However, there are still challenges that include massive follicle loss and short graft longevity after transplantation [8]. Therefore, there is still much to do to optimize the ovarian tissue cryopreservation procedure. Stromal cells are the main population of ovarian tissue and play significant roles in ovarian function and follicle development. Therefore, understanding the basic biophysical and cryobiological features of stromal cells should be helpful to develop the appropriate cryopreservation procedures. To our knowledge, this is the first study to provide this fundamental information by evaluating the OTLs of OSCs and the effect of osmotic stress on oxidant–antioxidant balance, mitochondrial function and stromal cell viability and function. These studies were then followed up with additional studies of the effect of the optimal dose of melatonin on detrimental effects of osmotic stress in the human and bovine OSCs were evaluated. This research reveals that OSCs are vulnerable to osmotic stress, which is one of the characteristics linked with CPA exposure during cryopreservation procedures. Our results show the OTLs for suspended and adherent stromal cells both in human and bovine are very narrow, or in other words, these stromal cells are particularly osmotically sensitive—much more than any other somatic cell type studied to our knowledge. With this very narrow osmotic window, it is understandable why previous reports showed that stromal cells are particularly vulnerable to the deleterious effects of cryopreservation, such as extracellular matrix deformity, necrosis, apoptosis with an irregularly shaped nucleus and cytoplasmic and plasma membrane abnormality [79,80]. To minimize the damaging effect of osmotic stress on OSCs, it is important to maintain OSC volume in this osmotic tolerance window [37,38].

Previous studies on different cell types indicated the importance of OTLs and the role of osmotic stress in actin filament disruption [36], membrane–cytoskeleton complex impairment [81], alteration of membrane phospholipid organization [54], modification of gene expression, changes to nuclear morphology [82] and apoptosis [83]. Our data revealed that deviation from iso-osmolality is associated with a significant decrease in the percentage of viable cells compared to the control group. These two variables show a very strong negative correlation. Although we did not evaluate apoptosis or necrosis in our experiment, our results are consistent with previous studies that showed osmotic stress directly alters cell and nuclear shapes [82,84] and subsequently influences cell survival by stimulating apoptosis and necrosis [85,86]. There are different possible signaling pathways that might be involved in cell death such as the mitogen-activated protein kinases family (MAPKs), a signal transduction pathway responsible for transferring the signals from the extracellular environment into the cells that plays a key role in cellular functions, such as cell survival [87]. In particular, osmotic stress has been reported to change the localization of a number of polarity proteins, including Scrib, which has been suggested to be intimately related to the control of MAPK kinase signaling [88]. While the activation of some members of this family, such as MEK1/2-ERK1/2 and phosphatidylinositol 3-kinase (PI3K)-Akt pathways, inhibit apoptosis [89], sustained activation of other members of the MAP kinases family, such as the c-Jun NH2-terminal kinases (JNKs) and the p38 mitogen-activated protein kinases (p38 MAPKs), followed by osmotic stress, inhibits cell cycle progression and induces apoptosis [90]. Another study showed that JNK1-1 and JNK1-2 are activated immediately by osmotic stress [91]. Moreover, following the activation of caspase-3, JNK1-2 is proteolyzed and can lead to the release of cytochrome c and then triggers caspase-3 activity. Indeed, this crosstalk produces a positive feedback loop [91].

An alternative or additional pathway that could be affected is the release of calcium. Ca^2+^ is increased by osmotic stress and it may cause the activation of the calpain protein (Ca^2+^ activated non-lysosomal cysteine proteases) that can accelerate apoptosis via the cleavage of the Bcl-2 family members and then triggers cytochrome c release and caspase-3 activity [89]. In hyperosmotic conditions, the other possible pathway is eIF2α phosphorylation caused by cell shrinkage, which is essential for transferring the RNA-binding protein hnRNP A1 from the nuclei to the cytoplasm. This protein inhibits the translation of anti-apoptotic genes and triggers pro-apoptotic genes [92].

Our results indicated that supplementation of MLT in anisotonic solutions can increase viability and decrease osmotic sensitivity of human and bovine stromal cells. A large body of evidence supports the positive effect of MLT addition to freezing media on cell viability and cell apoptosis during cryopreservation in different cell types [93,94]. Previously, Garcia et al. showed that MLT protects cell membranes from free radical attack by reducing oxidative stress and by optimizing electron transfer through the ETC. Melatonin also inhibits rigidity caused by ROS and conserves optimal fluidity levels in biological membranes [95], which possibly enhances the cell viability and decreases osmotic sensitivity. Possible involved signaling pathways in the antiapoptotic effect of melatonin include the MAPKs family, the Bcl-2/Bax complex and cardiolipin peroxidation. In particular, for the MAPK family, Luchetti et al. [96] reported that melatonin increases cell viability via improving phosphorylation and the activation of ERK 1/2 and inhibits the activation of the stress kinases p38 MAPK and JNK which are responsible for cell apoptosis. Additionally, it was reported that MLT stimulates mRNA transcription of antiapoptotic proteins BCL-2 and BCL-XL and inhibits the expression of the proapoptotic protein BAX and CAS-3 [97] which are responsible for cell viability and apoptosis. Finally, it has been proposed that melatonin prevents cardiolipin peroxidation in heart mitochondria, which is responsible for the mitochondrial permeability transition (MPT) induction and cytochrome c release [98]. Additionally, the PI3K/Akt and the JAK2/STAT3 signaling pathways were reported to play a role in the protective effects of melatonin against oxidative stress injury [99,100].

Another crucial aspect is the role of extra ROS in OSCs viability/apoptosis. Studies in other cell types indicated that an elevated level of ROS may result in mitochondrial impairment and activate the mitochondrial apoptotic pathway [101,102]. Briefly, induced superoxide anion generation caused by osmotic stress can trigger the intermembrane of mitochondria to release cytochrome c into the cytosol by the oxidative alteration of cardiolipin (acidic lipoprotein that is located mostly in the inner mitochondrial membrane), because of the increased level of ROS [103]. Then, cytochrome c activates the Apaf-1, caspase-9 and, finally, cell death/apoptosis [104]. In agreement with previous studies, [39,105] we found that OSCs under osmotic stress generate higher amounts of ROS, and ROS levels are negatively correlated with cell viability. Additionally, there is a strong positive correlation between osmotic stress and ROS production level. Burnaugh et al. showed, in sperm, that anisosmotic conditions can cause superoxide anion production and concomitant enhancement of protein tyrosine phosphorylation [39]. In the present work, the specific mechanism of ROS production in stromal cells caused by osmotic stress is not clear. However, based on the previous studies in other cell types, the explanation could be one of at least three possibilities. First, the osmotically-induced cell volume changes may trigger the membrane phospholipase A2 to cause an increase in the concentration of arachidonic acid (AA), which subsequently may stimulate the NADPH oxidase complex and then the production of superoxide anions [42]. Second, a study by Reinehr et al. showed that hypoosmotic conditions stimulate ROS generation in cultured astrocytes through serine phosphorylation of p47phox and it was inhibited by a NOX inhibitor [106]. Third, osmotic stress in skeletal muscle led to a calcium spark (elevated level of Ca^2+^ in the cytosol) as well as ROS formation [107]. Sarcoplasmic/endoplasmic reticulum or mechanosensitive/osmosensitive transient receptor potential vanilloid channels play roles in this calcium surge [107,108].

Based on the above findings, another explanation is that the osmotic stress may trigger the release of Ca^2+^ into the cytosol and subsequently Ca^2+^ may flow into mitochondria and induce NOX activity and ROS production. Both Ca^2+^ and ROS work as secondary messengers in cell signaling and cooperate bidirectionally. Indeed, ROS can control intracellular Ca^2+^, and enhanced Ca^2+^ levels can trigger a mitochondrial permeability transition and motivate ROS production. In agreement with previous studies, this study reveals that MLT reduced osmotic stress-induced ROS in human and bovine OSCs. This result is in agreement with previous reports [109,110], showing that MLT can easily cross the cell membranes and directly scavenge ROS and, because of its structure, each molecule of melatonin is able to scavenge four or more reactive species [72]. Another possible protective mechanism of melatonin is its ability to inhibit the release of AA followed by the reduction in the cPLA2 protein and mRNA expression [111]. Additionally, melatonin can inhibit the NADPH oxidase activation and reduce the ROS production via preventing the p47(phox) phosphorylation (subunit of NADPH oxidase) through a PI3K/Akt signalling pathway, inhibiting the translocation of the p47(phox) and p67(phox) subunit to the membrane, reducing the binding of p47(phox) to gp91(phox), and impairing the NADPH oxidase assembly [112].

This research reveals that osmotic stress might reduce total antioxidant levels in human and bovine OSCs in isotonic conditions and MLT can ameliorate negative effects of osmotic stress on antioxidant levels, which is in line with previous studies. Li et al. showed that in primary human corneal epithelial cells, hyperosmolality conditions reduced expression of the nuclear transcriptional factor (Nrf2) which is responsible for reduction in ROS levels and increasing the total antioxidant level via regulation of antioxidant gene expression, such as NAD(P)H quinoneoxido reductase 1 (NQO1), glutathione peroxidase (GPx) and glutamate cysteine ligase (GCLC) [113]. Similarly, another study showed that hyperosmolarity culture enhanced ROS production and decreased the total SOD activity compared with the control [114]; however, on the other hand, some studies have revealed that melatonin can regulate the expression of antioxidant enzymes through the activation of Nrf2 and its downstream genes such as HO-1, CAT, SOD and GSTM1 [28]. Towards this, Feng et al. showed that melatonin considerably increased the enzyme activity and protein expression of SOD, CAT and GSH-Px, which are the main antioxidant enzymes in cells and are responsible for converting superoxide radicals into water (SOD1 and SOD2: dismutase, the superoxide radical, into hydrogen peroxide and oxygen, and then GSH-Px and CAT convert the hydrogen peroxide to water) [94].

One of the main sites of ROS production in cells is mitochondria [115]. Previous studies showed that mitochondria play a critical role in nutrition metabolism and, as a result, in the production of energy [116]. The respiratory chain (RC) of the mitochondria produces energy by synthesizing adenosine triphosphate (ATP). It is made up of five multi-protein complexes (I to V) that are responsible for transferring electrons and converting superoxide to hydrogen peroxide. This reaction occurs along with the proton translocation from the matrix into the intermembrane space and the synthesis of ATP by complex V [117]. Electron leakage can occur anywhere along with the RC, resulting in the production of ROS. Complex I, III and then II, are the main source for ROS production in mitochondria [118]. In agreement with previous evidence in the literature, the results in this study showed that MRCA of human and bovine stromal cells decreases with increasing osmotic stress and it has a strong negative correlation with ROS levels as well as osmolality of the anisotonic solution. Previous work showed the abrupt rise in extracellular and subsequent cytoplasmic osmolality could directly trigger mitochondrial dysfunction through the alteration of the mitochondrial matrix volume [119,120]. This mitochondrial matrix volume change has a negative effect on respiratory chain function, mitochondrial proton gradient, cytochrome release and ATP generation [121]. It seems that the mTOR pathway plays a key role in this response [122]. Indeed, studies showed that osmotic stress-induced mitochondrial impairment is related to mitochondrial swelling, which can cause the opening of the mitochondrial permeability transition pore (PT-pore) and release of pro-apoptotic factors, apoptosis-inducing factor (AIF) and cytochrome C and superoxide leak followed by the disruption of the electron transport chain [122,123,124]. We found that melatonin can ameliorate the negative effect of osmotic stress on respiratory chain activity. However, this reduction was not significant in some human groups, possibly because of the method we used to calculate respiratory chain activity. In agreement with these results, other studies showed that melatonin can increase the activity of mitochondrial respiratory complexes I and IV [125,126]. One possible pathway could be through increasing the expression and activation of the mTOR pathway [127]. Moreover, as mentioned above, melatonin can prevent PT pore induction.

Although many studies have showed that melatonin is a powerful antioxidant and free radical scavenger, some studies suggest that melatonin might act as a pro-oxidant and promotes the generation of ROS in some cells [128,129], mostly in cancer cells [130,131]. In addition to cancer cells, the effects of high concentrations of melatonin on nontumor cells have also been reported [132,133]. While high levels of melatonin did not cause cytotoxicity in several cell types, it could inhibit cell proliferation, arrest cell growth and induce apoptosis in some nontumor cells [129,134]. The evidence suggests that the pro-oxidant action of melatonin is not correlated with cytotoxicity, but it is related to the melatonin concentration as well as cell type [130]. In agreement with these studies, our results showed that supplementation of anisotonic solutions with 10 mM melatonin is associated with higher ROS levels and lower cell viability in bovine OSCs. Wang et al. showed that, in the presence of the low concentrations of copper and polyphenol, melatonin increases ROS production through reducing copper. The addition of DNA to the system inhibits the reduction of copper by melatonin. On the other hand, their results revealed that, in the presence of high concentrations of copper and polyphenol, melatonin inhibits hydroxyl radical production and protects DNA by acting as a copper chelator [131].

Finally, our results indicate that OSCs show similar levels of sensitivity to osmotic stress in suspended and adherent groups, and the morphology of the cells related to the cell-cell/surface connection had no significant effect on osmotic tolerance. In agreement with our finding, it is reported that membrane hydraulic conductivity is similar between isolated and adherent cells, suggesting a small impact of the cytoskeletal structure on the water flow rate. In addition, this previous study indicated that the surface area involved with water transport in adherent PC-3 cells was almost the same as one in isolated cells. Therefore, the level and rate of dehydration is similar in PC-3 suspended and adherent cells [45]. On the other hand, Takagi et al. reported that adherent CHO cells were more susceptible to increased osmolalities compared to suspended cells because of the reduction in cell height and significant increase in the cell adhesion zone after hyperosmotic exposures, whereas there was no morphological change in the suspension cultures [46]. Last, but not least, in addition to the role of cell morphology (suspended vs adherent cell) on the level of damage during cryopreservation, the cell type and source should also be considered as critical factors [135,136].

## 5. Conclusions

In conclusion, in this study, the osmotic sensitivity of human and bovine OSCs was defined using cell viability. Our results show that a reasonably safe osmotic range for OSCs is approximately between 200 and 375 mOsm/kg for bovine and approximately between 150 and 500 mOsm/kg for human OSCs. This fundamental information explains why OSCs and, thus, ovarian tissue, are extremely sensitive to cryopreservation-induced damage. Moreover, the current study shows that osmotic stress can cause elevated levels of ROS generation and reduce the MRCA as well as antioxidant level and cell viability. Our results showed conclusively that exogenous melatonin can ameliorate some of the detrimental effects of osmotic stress. Since stromal cells have very narrow OTLs, these data are an important and useful tool for the rational optimization of the current protocol of OTC, allowing the functional preservation of enough stromal cells to enable post-graft follicle development and extended graft lifespan, and the addition of melatonin at the optimal concentration of 0.1 mM to ovarian tissue cryopreservation media is a promising option for clinical application.

## Figures and Tables

**Table 1:** A comparison of percentage of viability and ROS levels of suspended and adherent ovarian stromal cells in bovine and human after exposure to anisosmotic solutions supplemented with or without 0.1 mM of melatonin. All data are normalized mean ± standard error of the mean (SEM). Superscript b indicates *p* ≤ 0.01 vs. 300 mOsm/kg (control) group, superscript c indicates *p* ≤ 0.001 vs. 300 mOsm/kg (control) group, superscript d indicates *p* < 0.05 vs. non-treated counterpart group, superscript e indicates *p* ≤ 0.01 vs. non-treated counterpart group, superscript f indicates *p* ≤ 0.001 vs. non-treated counterpart group.

Bovine (*n* = 8)					Human (*n* = 3)				
	Suspended Cells		Adherent Cells			Suspended Cells		Adherent Cells	
	Control	Melatonin	Control	Melatonin		Control	Melatonin	Control	Melatonin
**mOsm**	**Viability**				**mOsm**	**Viability**			
25	0.57 ± 0.07 ^c^	0.72 ± 0.05 ^fc^	0.48 ± 0.03 ^c^	0.67 ± 0.03 ^fc^	25	0.68 ± 0.02 ^c^	0.78 ± 0.03 ^ec^	0.66 ± 0.0.2 ^c^	0.76 ± 0.03 ^dc^
150	0.68 ± 0.03 ^c^	0.84 ± 0.05 ^fc^	0.66 ± 0.02 ^c^	0.89 ± 0.05 ^fb^	150	0.8 ± 0.02 ^c^	0.93 ± 0.01 ^f^	0.79 ± 0.01 ^c^	0.95 ± 0.01 ^f^
500	0.66 ± 0.02 ^c^	0.85 ± 0.05 ^fc^	0.63 ± 0.03 ^c^	0.90 ± 0.08 ^fb^	500	0.81 ± 0.01 ^c^	0.94 ± 0.02 ^f^	0.78 ± 0.03 ^c^	0.90 ± 0.02 ^eb^
1000	0.42 ± 0.03 ^c^	0.56 ± 0.03 ^fc^	0.34 ± 0.02 ^c^	0.49 ± 0.02 ^fc^	1000	0.65 ± 0.01 ^c^	0.76 ± 0.02 ^ec^	0.53 ± 0.03 ^c^	0.74 ± 0.02 ^fc^
	**ROS Levels**					**ROS Levels**			
25	2.54 ± 0.06 ^c^	1.92 ± 0.08 ^fc^	2.63 ± 0.1 ^c^	2.06 ± 0.12 ^fc^	25	1.85 ± 0.05 ^c^	1.64 ± 0.03 ^dc^	1.65 ± 0.03 ^c^	1.43 ± 0.02 ^fc^
150	1.91 ± 0.03 ^c^	1.35 ± 0.04 ^fc^	1.57 ± 0.03 ^c^	1.23 ± 0.05	150	1.69 ± 0.09 ^c^	1.43 ± 0.03 ^ec^	1.44 ± 0.02 ^c^	1.25 ± 0.02 ^fc^
500	1.97 ± 0.13 ^c^	1.37 ± 0.07 ^fc^	1.89 ± 0.02 ^c^	1.40 ± 0.03 ^eb^	500	1.97 ± 0.02 ^c^	1.56 ± 0.03 ^fc^	1.70 ± 0.03 ^c^	1.20 ± 0.04 ^fc^
1000	2.51 ± 0.06 ^c^	1.87 ± 0.05 ^fc^	2.46 ± 0.08 ^c^	2.04 ± 0.1 ^dc^	1000	2.81 ± 0.05 ^c^	2.21 ± 0.3 ^fc^	2.66 ± 0.03 ^c^	2.11 ± 0.03 ^fc^

**Table 2:** A comparison of percentage of viability and ROS levels of suspended and adherent ovarian stromal cells in bovine after exposure to anisosmotic solutions supplemented with different concentrations of melatonin. All data are normalized mean ± standard error of the mean (SEM). (a) *p* < 0.05 vs. 300 mOsm/kg group, (b) *p* ≤ 0.01 vs. 300 mOsm/kg group, (c) *p* ≤ 0.001 vs. 300 mOsm/kg group, (d) *p* < 0.05 vs. non-treated counterpart group, (e) *p* ≤ 0.01 vs. non-treated counterpart group, (f) *p* ≤ 0.001 vs. non-treated counterpart group.

Bovine (*n* = 8)												
Suspended Cells							Adherent Cells					
	Control (Melatonin 0 mM)	Melatonin 0.001 mM	Melatonin 0.01 mM	Melatonin 0.1 mM	Melatonin 1 mM	Melatonin 10 mM	Control (Melatonin 0 mM)	Melatonin 0.001 mM	Melatonin 0.01 mM	Melatonin 0.1 mM	Melatonin 1 mM	Melatonin 10 mM
**mOsm**	**Viability**											
25	0.50 ± 0.01 ^c^	0.63 ± 0.01 ^fc^	0.64 ± 0.05 ^fc^	0.65 ± 0.05 ^fc^	0.51 ± 0.05 ^c^	0.20 ± 0.01 ^fc^	0.54 ± 0.03 ^c^	0.57 ± 0.03 ^c^	0.64 ± 0.04 ^c^	0.70 ± 0.04 ^fc^	0.55 ± 0.03 ^c^	0.16 ± 0.02 ^fc^
150	0.66 ± 0.1 ^c^	0.70 ± 0.02 ^c^	0.76 ± 0.03 ^c^	0.85 ± 0.02 ^fc^	0.71 ± 0.01 ^c^	0.24 ± 0.02 ^fc^	0.66 ± 0.04 ^c^	0.69 ± 0.03 ^c^	0.76 ± 0.02 ^c^	0.89 ± 0.02 ^f^	0.75 ± 0.02 ^c^	0.20 ± 0.03 ^fc^
300	1 ± 0	1.01 ± 0.01	1.05 ± 0.01	1.09 ± 0.01	0.87 ± 0.02 ^e^	0.72 ± 0.02 ^f^	1 ± 0	1.01 ± 0.01	1.04 ± 0.01	1.09 ± 0.02	0.92 ± 0.04	0.36 ± 0.03 ^f^
500	0.65 ± 0.03 ^c^	0.66 ± 0.01 ^c^	0.68 ± 0.06 ^c^	0.83 ± 0.06 ^fc^	0.75 ± 0.05 ^dc^	0.67 ± 0.03 ^c^	0.63 ± 0.04 ^c^	0.67 ± 0.03 ^c^	0.74 ± 0.04 ^c^	0.90 ± 0.06 ^f^	0.74 ± 0.04 ^c^	0.33 ± 0.03 ^fc^
1000	0.42 ± 0.03 ^c^	0.36 ± 0.02 ^c^	0.42 ± 0.03 ^dc^	0.49 ± 0.03 ^fc^	0.37 ± 0.03 ^c^	0.19 ± 0.03 ^fc^	0.33 ± 0.02 ^c^	0.43 ± 0.02 ^c^	0.53 ± 0.02 ^c^	0.56 ± 0.01 ^dc^	0.45 ± 0.01 ^c^	0.25 ± 0.02 ^c^
**mOsm**	**ROS Levels**											
25	2.45 ± 0.07 ^c^	2.22 ± 0.07 ^c^	1.82 ± 0.02 ^fc^	1.86 ± 0.08 ^fc^	2.06 ± 0.09 ^fc^	2.31 ± 0.05 ^c^	2.60 ± 0.05 ^c^	2.39 ± 0.09 ^c^	2.10 ± 0.10 ^ec^	2.04 ± 0.04 ^fc^	2.23 ± 0.12 ^c^	2.75 ± 0.29 ^c^
150	1.94 ± 0.04 ^c^	1.75 ± 0.06 ^c^	1.37 ± 0.07 ^fc^	1.35 ± 0.03 ^fb^	1.65 ± 0.01 ^c^	1.98 ± 0.04 ^c^	1.62 ± 0.02 ^c^	1.45 ± 0.07 ^a^	1.10 ± 0.02 ^e^	1.19 ± 0.02	1.41 ± 0.07	1.74 ± 0.12 ^c^
300	1 ± 0	0.90 ± 0.01	0.73 ± 0.02	0.78 ± 0.04	1.00 ± 0.06	1.38 ± 0.03 ^f^	1 ± 0	0.92 ± 0.02	0.74 ± 0.02	0.70 ± 0.03	0.90 ± 0.03	1.21 ± 0.02
500	2.01 ± 0.03 ^c^	1.78 ± 0.06 ^c^	1.39 ± 0.04 ^fc^	1.39 ± 0.02 ^fc^	1.79 ± 0.02 ^c^	1.96 ± 0.05 ^c^	1.92 ± 0.02 ^c^	1.73 ± 0.03 ^c^	1.43 ± 0.07 ^d^	1.48 ± 0.07 ^da^	1.67 ± 0.06 ^c^	1.92 ± 0.04 ^c^
1000	2.45 ± 0.03 ^c^	2.20 ± 0.08 ^c^	1.78 ± 0.06 ^fc^	1.86 ± 0.01 ^fc^	2.15 ± 0.01 ^dc^	2.35 ± 0.02 ^c^	2.47 ± 0.03 ^c^	2.24 ± 0.04 ^c^	1.93 ± 0.06 ^ec^	1.97 ± 0.02 ^ec^	2.16 ± 0.06 ^c^	2.43 ± 0.03 ^c^

## Data Availability

Data is contained within the article.
